# Tungsten Toxicity in Plants

**DOI:** 10.3390/plants1020082

**Published:** 2012-11-16

**Authors:** Ioannis-Dimosthenis S. Adamakis, Emmanuel Panteris, Eleftherios P. Eleftheriou

**Affiliations:** Department of Botany, School of Biology, Aristotle University of Thessaloniki, 541 24 Thessaloniki, Greece; Email: iadamaki@bio.auth.gr (I.-D.S.A.); epanter@bio.auth.gr (E.P.)

**Keywords:** actin microfilaments, endoplasmic reticulum stress, unfolded protein response, microtubules, mitosis, molybdenum cofactor, programmed cell death, tungsten toxicity

## Abstract

Tungsten (W) is a rare heavy metal, widely used in a range of industrial, military and household applications due to its unique physical properties. These activities inevitably have accounted for local W accumulation at high concentrations, raising concerns about its effects for living organisms. In plants, W has primarily been used as an inhibitor of the molybdoenzymes, since it antagonizes molybdenum (Mo) for the Mo-cofactor (MoCo) of these enzymes. However, recent advances indicate that, beyond Mo-enzyme inhibition, W has toxic attributes similar with those of other heavy metals. These include hindering of seedling growth, reduction of root and shoot biomass, ultrastructural malformations of cell components, aberration of cell cycle, disruption of the cytoskeleton and deregulation of gene expression related with programmed cell death (PCD). In this article, the recent available information on W toxicity in plants and plant cells is reviewed, and the knowledge gaps and the most pertinent research directions are outlined.

## Abbreviations

AO: aldehyde oxidase
ER: endoplasmic reticulum
mARC: mitochondrial amidoxime reducing component
MoCo: molybdenum cofactor
Mo-enzymes: molybdoenzymes
MT: microtubule
NR: nitrate reductase
PCD: programmed cell death
ROS: reactive oxygen species
SO: sulphite oxidase
UPR: unfolded protein response
W-PCD: tungsten-induced programmed cell death
XDH: xanthine dehydrogenase

## 1. Introduction

Tungsten (W) is a rare transition element, belonging in the Group VI-B of the Periodic Table of elements, along with chromium, molybdenum (Mo) and seaborgium. Due to its unique physical and chemical properties, W is an extremely useful element having broad industrial, civil and military applications, ranging from daily household goods such as light bulbs and golf clubs, to electronics, hard metal tools, munitions and highly specialized components of advanced modern science and technology [1]. Although W is present in the soil in traces, its concentration can be very high locally due to anthropogenic activities, such as active and abandoned mining sites [2,3,4]. The use in agriculture of phosphate fertilizers that may contain W up to 100 mg kg^−1^ [5] and the replacement of lead with W in military applications and war practices, e.g., in projectiles, as an environmentally “benign” alternative, have increased surface soil W concentration [6,7] and the potential exposure of humans to W [8]. Post-consumer discharges also contribute to the flow of W in the environment. Although the recycling rates of the metal discharges reach 50%–70%, it has been pointed out that there still exists a possibility for sustainable use and/or better control in the environmental emissions of W [9].

Unlike many other metals, such as copper, cadmium, nickel, aluminium and mercury, which have been well documented as toxic and harmful to living organisms, the existing knowledge on the presumed toxicological profile of W is relatively limited and constitutes a subject of productive discussion within the scientific community [1,10]. Hence, W has been considered either as a non-toxic or non deleterious metal [11] or it has been regarded that it might be tumorous and leukemogenic in animal cells [12].

While in the former USSR investigations concerning the toxicological profile of W have began already by the 1950s and environmental regulations on W pollution were developed since the 1980s, research on W in USA and the European Union has started relatively recently [13]. Commence of research over W toxicology in the USA was encouraged due to cases of the leukemia clusters in Nevada State [14]. Especially the cluster in the city of Fallon, regarded as “one of the most unique …ever reported” [15], provoked extensive research in an attempt to find the preliminary cause. Despite the high levels of W, as well as of arsenic, JP-8 jet fuel and pesticides found in the area, no direct relationship between leukemia and W exposure was found, while no environmental exposure that could explain the cluster of childhood leukemia was ever discovered (among others see [16]). However, due to a lack of experimental data on which regulatory decisions could be established, the National Institute of Health classified W as a priority chemical for toxicological research [16]. In 2008, W was characterized as a substance of concern and an emerging pollutant [13]. A question then arises whether W could be considered as an environmental problem, boosting relevant research over recent years, mostly on microbes, animals and humans ([17,18,19,20,21,22,23,24]; and references therein).

In plants, the relevant information so far refers mainly to the molybdoenzyme (Mo-enzyme) research, where W is used as an inhibitor of Mo-enzymes (Section 2.2). However, recent reports indicate that, apart from its effects on the Mo-enzymes, W inhibits growth, deregulates cell division, disrupts cortical microtubule (MT) integrity and function, and induces programmed cell death (PCD) [25,26,27,28]. Consequently, W toxicity to plants should be analyzed under a broadened view, not solely as a Mo-enzyme inhibitor but also as a heavy metal with further effects [29]. Therefore, this review aims to: (i) recapitulate the existing information on W effects on the Mo-enzymes, (ii) summarize W effects on plant growth, cytology and genome expression under W stress, and (iii) outline the potential mechanisms by which W exerts its effects beyond Mo-enzyme inhibition. As such, this review should be considered as a complement to the few existing reviews in plants [29] and the many more in other organisms (e.g., [10,17,30,31,32,33]).

## 2. W Effects on Plants

### 2.1. W Uptake and Accumulation

W is uptaken by the roots and translocated to the upper plant organs via the xylem. W accumulation in the root tissues is much higher than in the shoot [4,34,35]. Anthocyanins may also facilitate W accumulation in some plant species [36]. Plants growing on abandoned mines that are heavily contaminated with several metals and metalloids including W may accumulate metals and evolve tolerance against these pollutants. For instance, plants of *Calluna vulgaris *naturally growing in an abandoned early twentieth century W mine in UK accumulated W in the root tissues at 56% of the total amount of W calculated in the spoil (1,637 mg kg^−1^) separated from the rhizosphere [4]. Ryegrass is another plant able to accumulate W; its seedlings germinated in soil amended with high concentrations of metallic W (10,000 mg kg^−1^) accumulated significant amounts of the metal in the leaves (13,535 ± 6,125 mg kg^−1^) after two months [19]. Many more plant species have been indentified having the potential of W accumulation, some of which are *Digitalis purpurea*, *Chamaespartium tridentatum*, *Cistus ladanifer*, *Pinus pinaster*, *Erica umbellata* and *Quercus ilex* subsp. *ballota* [3]. Except natural outcrops, cultivated plants have been shown to uptake W and were used for W and cobalt removal from wastewaters when applied to agricultural fields [37,38]. In particular, cabbage and carrot fields were irrigated with wastewater (or 50% wastewater diluted with unpolluted water) from a W and cobalt processing unit. These experiments have indicated that migration of the metals to the soil is limited when plants are grown in the field as compared to fields without plants. W-tolerant plant species may be useful in mine stabilization and re-vegetation strategies, while W-accumulating ones can potentially be applied in phytoremediation-based technologies for the cleanup of W-contaminated soils [3,4,19,34]. Accordingly, deeper knowledge about the effects of W on plants and the pathway W ions use in entering the cell is essential.

### 2.2. W-Mo Antagonism: W as an Inhibitor of Mo-Enzyme Activity

**Figure 1:**
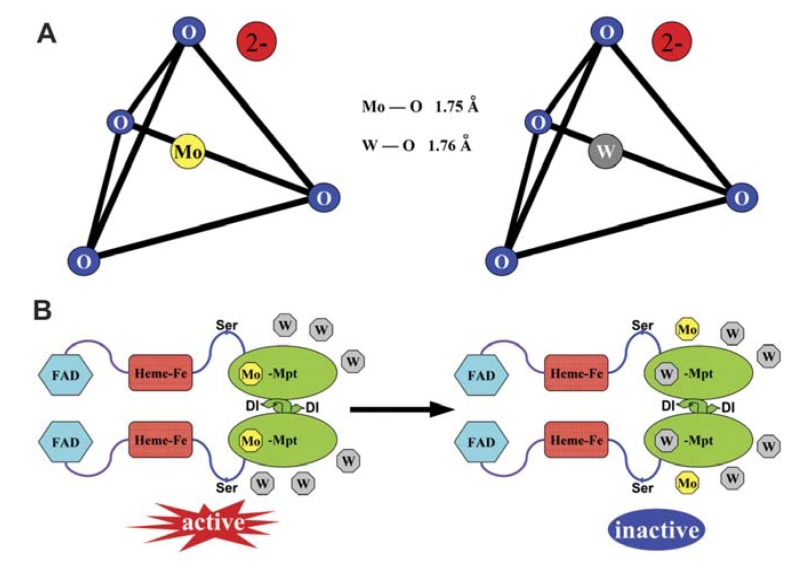
A comparison of tungstate and molybdate structure that underlies their antagonism for molybdenum cofactor (MoCo). (**A**) Structural schemes of tungstate and molybdate. The metal atoms are at the centre of a tetrahedron of oxygen atoms. (**B**) Tungstate competes with molybdate for incorporation into the MoCo, resulting in inactivation of nitrate reductase (NR). FAD: flavin adenine dinucleotide, Mo-Mpt: molybdenum-molybdopterin. Reprinted from [29], with kind permission from both the authors and Oxford University Press.

As soon as W enters the plant cell it may cause some adverse effects on specific targets. Of the first targets indentified were the Mo-enzymes, mostly due to W-Mo antagonism. Over evolution, due to their similar physical and chemical properties (Figure 1A), W and Mo have been incorporated into the active sites of key enzymes, highly broadening their catalytic diversity in the biological systems. In the hot and anaerobic conditions under which life probably arose, physicochemical properties of elements accounted so that W might have been first acquired by the early life forms than Mo. As the earth’s crust cooled and the atmosphere became aerobic, Mo presumably substituted W in the enzyme active sites [30]. Nowadays, the consensus is that Mo is required by most living organisms and the Mo-activated enzymes are encountered in all aerobic ones, whereas W occurs in the obligate, typically thermophilic anaerobes [17,30,39]. Otherwise, W is not necessary for eukaryotes and no “tungsto-enzymes” have so far been identified in them. The W-Mo antagonism for the same enzyme site has been inherited today when W occurs in high concentrations. This was already stated by 1956 in *Asprergillus nidulans*, where Higgins *et al*. [40] noticed that when nitrate was the sole nitrogen source, tungstate in a molar ratio of 20:1 to Mo readily induced growth inhibition.

Within the cell, W antagonizes effectively and may substitute Mo in the molybdenum cofactor (MoCo) of the Mo-enzymes (Figure 1B) [29]. Mo-enzymes are required for diverse key reactions in carbon, sulphur and nitrogen metabolism. In plants, they can be subdivided into two families: the xanthine oxidase family, represented by xanthine dehydrogenase (XDH) and aldehyde oxidase (AO); and the sulphite oxidase (SO) family, represented by SO and nitrate reductase (NR). Recently, the mitochondrial amidoxime reducing component (mARC) was also identified, but it has not yet been integrated into one of the aforementioned families [41].

Omarov *et al*. [42] reported that application of 250 μΜ of tungstate resulted in AO inhibition in barley roots and leaves. The authors used the inhibitory effect in order to conclude that Mo is involved in the catalytic centres of the AO. Moreover, in W-treated *Zea mays* plants or in plants grown in W-enriched soils, AO activity appeared significantly reduced [43]. In *Zea mays*, inhibition of drought-induced abscisic acid synthesis was noticed and it was suggested that the MoCo of AO may be important for the drought-induced abscisic acid biosynthesis. Similar reduction of AO activity was noticed in barley, when treated with increasing tungstate concentrations [44]. Also, the activity of XDH was sensitive to W. In barley roots, 100 and 500 μΜ tungstate in the nutrient medium drastically reduced XDH activity; again, the drop in enzyme activity was attributed to the reduction of the MoCo [44]. AO, XDH and SO have the ability to normally produce hydrogen peroxide (H_2_O_2_) [41]. Recently, it was found that tungstate inhibited the H_2_O_2_-generating activity of both SO and AO [45]. Regarding mARC, as far as we are aware no reports about the effect of W on this enzyme exist yet.

Plenty of studies have demonstrated the inhibitory action of W to NR activity, and in the form of sodium tungstate (Na_2_WO_4_) it was used as a casual inhibitor of NR [46,47]. So, since W could be easily applied, it was and is still used as a NR inhibitor in plant NR-research, in order to lower the production of nitric oxide (NO) (among others see [29,46,47,48]), since the NR-dependent NO formation is the best-characterized NO source in plants [49].

However, some researchers have questioned the ability of tungstate to affect NO production. Characteristic examples are the studies by Xiong *et al*. [50] and Wang *et al*. [51], who have reported that 100 μΜ of tungstate had no effect on endogenous NO concentration in rice and lupin, respectively. Moreover, tungstate inhibited primary and crown root elongation in rice plants [50]. So an assumption was made that tungstate could be toxic to plant roots and that tungstate-induced inhibition on root elongation does not correlate with endogenous NO content. More recently, Kumar *et al.* [52], while investigating the generation of NO in isolated protoplasts and chloroplasts of *Brassica napus*, found that NO production was not inhibited by 250 μM Na_2_WO_4_. Consequently, it comes as no surprise the question: “Tungstate: is it really a specific NR inhibitor in plant NO research?” [29].

Since there are doubts on the ability of tungstate to affect NO, and tungstate’s toxicity in roots could not be only due to NO inhibition, a question that unavoidably arises is: are Mo-enzymes the only targets of W toxicity? Recent experimentation revealed further subcellular targets, correlated with defects in root growth and morphology.

### 2.3. W Is More Than a Mo-Enzyme Inhibitor

#### 2.3.1. W Inhibits Plant Growth

The first visible symptom of W toxicity was a general reduction of root and shoot growth. This has been observed in different plant species and some of the results are summarized in Table 1. Except for a reduction in plant growth or biomass production, notable is the case of W application in *Brassica rapa*, *B. juncea* and *B. oleracea*, where it caused the accumulation of a blue complex in the roots [36].

**Table 1:** Macroscopic effects of W in higher plants.

Plant species	Treatments	Effects/Results	Reference
*Brassica* * rapa*	Seeds sown in Magenta boxes on ½ MS medium with 50-150 mg L^−1^ W as Na_2_WO_4_.	Increased production of a blue substance correlated with anthocyanin accumulation.	[36]
*B. juncea*			
*B. oleracea*			
*Hordeum vulgare* L. cv. Steptoe (barley)	0.25, 0.5, 1, 10, 100, 500 μM Na_2_WO_4_, 9 days. Hydroponics.	Significant reduction in root and shoot biomass at high concentrations (≥10 μM).	[44]
*Lolium perenne *(ryegrass)	Urban and forest soil amended with ammunition grade W powder (average particle size 5 μM): 1, 10, 100, 1,000, 10,000 mg kg^−1^. Up to 9 months.	Serious reduction of plant growth and death after two months.	[19]
*Pisum sativum* cv. Onmard (pea)	200, 500 mg L^−1^ Na_2_WO_4_, up to 8 days. Hydroponics.	Inhibition of root elongation and lateral root formation, retardment of seedling growth rate and new leaf emergence.	[25]
*Gossypium hisutum* cv.			
Campo (cotton)			
*Helianthus annuus* L. (sunflower)	Processed field soil, spiked with 6,500 mg kg^−1^ metallic W powder, aged for six months. Diluted soil samples contained from 0 to 6,500 mg W kg^−1^. Plants grown for two or four weeks.	Plant total weight significantly reduced at W concentration ≥2,600 mg kg^−1^, root and shoot length reduced at ≥3,900 mg kg^−1^.	[34]
*Avena sativa* cv. Ogle (oat)	Natural soil containing 0 (control), 0.803, 2.41, 7.21, 21.7, 65.0, 195.1, and 586 mg W kg^−1^ dry soil.	Toxicity was recorded at ~58.6-293 mg W kg^−1^ dry soil, with lettuce being more sensitive than radish, followed by oat.	[53]
*Raphanus sativus* cv. Crimson Giant			
(radish)			
*Lactuca sativa* cv. Grand Rapids (lettuce)			
*Triticum aestivum* L. var Raj4037.	Potted soil watered with 3, 9, 27, 81, 243 mg kg^−1^ Na_2_WO_4_.2H_2_O solutions plus control. Samples harvested after 60 days.	Lower concentrations (3, 9 mg kg^−1^) of W had promotive effects in growth, biomass, chlorophyll, carbohydrate and Mo contents, higher ones decreased them.	[35]
*Vigna unguiculata* L. Walp. var. Sephali Shikha-313	Potted soil watered with 5, 10, 15, 20, 25 μg g^−1^ Na_2_WO_4_.2H_2_O solutions. Samples harvested after fruiting.	Lower applied doses (5, 10, 15 μg g^−1^) of W promoted root-shoot length. Higher doses retarded root-shoot length.	[54]
*Brassica oleracea* (cabbage)	Grown in aged W powder-spiked soil containing monomeric and polymeric tungstates provided as Na_2_WO_4_·2H_2_O.	Cabbage growth was impaired at 436 mg W kg^−1^ W soil.	[23]
*Lactuca sativa* (lettuce)			

However, the mechanism by which W exerted this effect has not been extensively studied, and commonly visible defects were correlated with the decreased activity of the Mo-enzymes in response to increasing amounts of tungstate [29,43]. Several further assumptions have been made. W could affect plants indirectly, such as by lowering soil pH [53], which alters the availability of nutrients in the soil. For instance, W is oxidized to tungstate in soil and in turn it can be polymerized with phosphate, depleting the latter from the soil [55].

Another indirect mechanism of toxicity may be the alteration of soil microflora necessary for plant survival. Reports on W effects on microbes have shown that W can either be beneficial or detrimental for the microbial biomass. For example, Strigul *et al*. [19] reported a statistically significant decrease in microbial biomass in *Bacillus subtilis* and *Pseudomonas fluorescens*. The above study showed that the presence of metallic W resulted in the death of a substantial portion of the bacterial component of the soil community and an increase of fungal biomass. On the other hand, Rigelberg *et al*. [20] reported a general positive effect of W on soil microbial biomass, as revealed by the increase in Gram-negative and Gram-positive bacterial fatty acids at relatively low W concentrations and negative effects at higher concentrations. Of the most important soil bacteria affected by W are the nitrogen-fixing ones, as for example the *Azotobacter vinelandii* [20]. W may also affect the N_2_-reducing capacity of these bacteria by substituting Mo in nitrogenase, as it was found in *Rhodobacter capsulatus* [56].

W could also affect root growth directly by targeting specific cellular components or biochemical pathways (besides the Mo-enzymes). The effects of W on phosphate-dependent biochemical pathways have been already examined in animal cells [57] and remain yet to be studied in plants. For instance, W may disturb phosphate concentration inside the cell, altering phosphate homeostasis within the tissues, disrupting phosphorylating reactions including cell signalling pathways or even production of adenosine triphosphate (ATP). Moreover, inhibition of root elongation may be related with ultrastructural defects caused by W in root cells [25,26,28]. Root elongation is achieved by both cell division and expansion [58], both of which are reported to be affected by W.

Recent reports maintain that W, like other heavy metals, affected nucleus morphology and cell division [25,28]. In particular, the nuclei of W-treated root cells in *Pisum sativum* and *Gossypium hirsutum* seedlings appeared larger and frequently multi-lobed, as compared to the rounded and smooth ones of untreated samples. The chromatin of W-affected nuclei became condensed and peripherally distributed, and in some cases atypical double membrane structures occurred in the nucleoplasm defining chromatin condensation in sub-peripheral compartments. Cytoplasmic components such as ribosomes, mitochondria, membranes, vesicles or small vacuoles and lipid droplets, were frequently entrapped within the nucleoplasm of W-affected nuclei. These intranuclear entrapments imply a defect in cell division. Moreover, nucleoli of W-affected cells were more prominent and contained bigger nucleolar vacuoles than those of untreated ones. Also, in W-treated roots incomplete, misaligned or unilaterally extended cytokinetic walls were readily recognized. These cytokinetic aberrations were most likely attributed to defective MT polymerization and function, as phragmoplast MTs were reduced in number [25,28]. There are many reports stating that toxic metal ions produce several defects in dividing plant cells, including abnormal mitoses, decreased mitotic index, alterations of nucleus and nucleolus morphology, cell cycle aberrations and DNA defects [59]. Similarly, evidence has been reported that W affects plant DNA: W particles caused a breakage of phosphodiester bonds in native DNA at a limited number of sites in wheat embryos after a biolistic transformation [60].

A common target of W toxicity among different plant species are the cortical MTs, as this was revealed by exposing taxonomically diverse land plant taxa representing monocots (*Allium cepa*, *Zea mays*), dicots (*Arabidopsis thaliana*), gymnosperms (*Pinus brutia*), pterophytes (*Adiantum capillus-veneris*) and bryophytes (*Physcomitrella patens*, *Tortula muralis*), to aqueous Na_2_WO_4_ solutions [27]. As cortical MTs are of critical importance for cell shaping and anisotropic elongation, disruption by W could also be responsible for root growth inhibition [26], which is a general trait of heavy metal and metalloid toxicity ([61,62]; and references therein). Although the mechanism by which W induced this effect is unclear, it seems to be a side effect of a more prominent response, probably involving programmed cell death (PCD) processes [28].

#### 2.3.2. W-Induced Programmed Cell Death (PCD)

One of the main responses of plants to developmental and biotic/abiotic environmental stimuli is the execution of cell “suicide” or programmed cell death (PCD) [63,64]. PCD is a genetically determined process found throughout animal and plant kingdoms, involving the actively controlled and precise degradation of cellular components, aiming at selective elimination of harmful, unwanted or damaged cells in eukaryotes [64]. Undesirable PCD may also be instigated by many biotic agents such as pathogens [65] and abiotic factors like extreme temperatures [66]. Different types of PCD in plants have been recognized, all sharing a unique process that depends on vacuole collapse, which releases sequestered hydrolases that degenerate cell components and lead to cell death [67,68,69]. Cleavage of nuclear DNA into oligonucleosomal fragments (DNA laddering) is a potential indicator of PCD in plants [63,70,71]. Plant developmental PCD (e.g., the differentiation of tracheary elements of xylem) has been intensively studied and in most cases it is triggered by plant hormones [64,65,67,69].

The execution of a kind of W-induced PCD in plants was recently documented for the first time in root tip cells of *Pisum sativum* exposed to 200 mg L^−1^ Na_2_WO_4_ for 12, 24, 48 and 72 h [28], rendering cell death a common feature of W toxicity occurring in both plants and animals [21,22]. However, W-induced PCD (W-PCD for brevity) has been stated to exert certain peculiarities [28], therefore its features are being compared with those of other metal-induced PCD cases, which are summarized in Table 2.

**Table 2:** Programmed cell death (PCD) induced by toxic metals in plants.

Metal	Plant species	Conditions and responses	Reference
Cd	*Nicotiana tabacum* Mill.	Chronic exposure of suspension cells to 50-100 mM CdSO_4_ induced apoptotic-like PCD, including DNA fragmentation into oligonucleosomal units (50-200 kb fragments).	[72]
	BY-2 cell line		
Al	*Hordeum* * vulgare*	In root-tip cells 0.1-1.0 mM Al treatments for 8 h induced PCD, possibly via a ROS-modified signal transduction pathway, whereas 10-50 mM Al treatments caused necrosis.	[73]
Cd	*Lycopersicon esculentum* Mill. (tomato). Cell line MsK8	Cadmium (CdSO_4_) induced apoptotic-like PCD that required increased H_2_O_2_ production and activation of phospholipase C and D and ethylene signalling pathways.	[74]
Al	*Lycopersicon esculentum* Mill. (tomato). Cell line MsK8	Suspension cells treated with 100 μM AlCl_3_ showed typical features of PCD (nuclear and cytoplasmic condensation), executed by caspase-like proteases.	[75]
Cd	*Lycopersicon esculentum* Mill. (tomato). Cell line MsK8	0.1 mM CdSO_4_ in cell suspension culture induced cell death after 24 h, involving caspase-like proteases, indicating that PCD took place.	[76]
Cd	*Nicotiana tabacum *L. (tobacco) BY-2 cell line	50 μM CdSO_4_ induced internucleosomal DNA fragmentation connected with the action of cysteine proteases and the loss of membrane integrity, in particular of tonoplast.	[70]
W	*Pisum sativum* L. cv. Onmard (pea)	Root tip cells of young seedlings exposed to 200 mg L^−1^ Na_2_WO_4_ for 12-72 h executed PCD through ER stress-UPR. The expression of the PCD-related genes *DAD-1* and *HSR203J* was altered.	[28]

DNA laddering did not seem to be induced by W but only a slight DNA smearing, in contrast to what was observed in the case of cadmium [72] and aluminium [73]. DNA laddering is however a hallmark of the majority but not of all the cases of cells that undergo PCD [70]. Similarly to other metal-induced PCD phenomena, W-PCD appears to involve the participation of caspase-like enzymes (cf. [28], [75,76]). Caspase-like proteases or metacaspases, which have a common substrate with caspases, were found to participate in plant PCD [77,78]. Caspase inhibitors have been used to prevent the action of the above enzymes [78,79]. The pan-caspase inhibitor Z-VAD-FMK has been applied to prevent various kinds of PCD in plant cells [80,81], and it was indicated that caspase-like proteases might participate in W-PCD [28]. Moreover, the involvement of the 26S proteasome in W-PCD was indirectly shown in W-treated roots. Inhibition of the 26S proteasome activity by MG132 (Z-Leu-Leu-Leu-al) reduced considerably the effects of W on the W-affected cells, thus preventing W-PCD. Considering that the 26S proteasome is known to exhibit some caspase-like activity both in animal or yeast cells [82] and plant cells [83], it was concluded that the 26S proteasome is involved in W-PCD in plant cells [28].

How is this W-PCD being triggered? Aluminium-induced PCD is initiated by reactive oxygen species (ROS) production [73]. Low doses of ROS can induce the production of antioxidant enzymes, but when the concentration of ROS reaches a certain threshold a signal transduction pathway that results in PCD is activated, while high doses of ROS result in necrosis [84]. Similar evidence was obtained in cadmium-induced PCD, where the oxidative stress appeared to be “instrumental” in cell death [74]. In fact, redox changes are sensed by the plant cell as a “warning” message and, depending on the situation, genetic programs leading to stress acclimation or PCD are switched on [85]. Information about the participation of ROS signals during W application is currently lacking in plants and could be the objective of a future study. ROS induction has been already observed in mammalian cells treated with W-based nanoparticles [24].

In animal cells, heavy metals could lead to apoptosis through an endoplasmic reticulum (ER) stress response ([86]; and references therein). ER-stress is also found to be associated with a PCD-triggering pathway in plants [87,88]. Adamakis *et al*. [28] showed that W caused *DAD-1* silencing in W-treated roots of *Pisum sativum*. *DAD-1* encodes a subunit of oligosaccharyltransferase, which participates in *N*-linked protein glycosylation in the ER, a post-transcriptional modification fundamental for proper protein folding [89]. Inhibition of *N*-linked protein glycosylation, as for example with tunicamycin [90], results in the accumulation of unfolded proteins in the ER lumen, initiating the unfolded protein response (UPR), which in turn triggers PCD [88,91]. *DAD-1* silencing along with increased expression of three genes closely related with ER stress and UPR (*BiP-D*, *bZIP28* and *bZIP60*) and additional indirect supportive evidence derived from co-treatments of W and 4-phenylbutyric acid (a chemical suppressor of ER stress), were all consistent with the general conclusion that W induces a kind of PCD in plant cells triggered by the ER stress-UPR pathway [28]. The details, however, of the mechanisms involved are obscure and remain to be further disclosed.

### 2.4. A Model for W Entrance in Plant Cells

To sum up, besides Mo-enzyme inhibition W appears to induce a wide range of effects after it enters the plant cells. But how does W enter plant cells? Could it use the same entrance pathway as Mo does? The already described subcellular effects and additional pharmacological evidence [26] permit the proposal of a hypothetical model for W uptake depending on the entrance pathway of Mo, as is discussed below.

The uptake of Mo (as molybdate anion, MoO_4_^2^^−^) in plant cells starts with the attachment of Mo to a plasma transmembrane anion transporter. The available data are not clear on the identity of this transporter, although two proteins (MOT1 and MOT2) were identified in *Arabidopsis thaliana* as Mo transporters. However, none of them was found to be located in the plasma membrane [92]. After its entry, Mo interacts with the Cnx1 protein, which is bound to the actin microfilaments of the cortical cytoplasm [93], in the same way as its mammalian homologue gephyrin binds to tubulin [94]. Cnx1 may interact with a transmembrane anion transporter to facilitate the Mo transport towards molybdopterin to form the MoCo [93].

**Figure 2:**
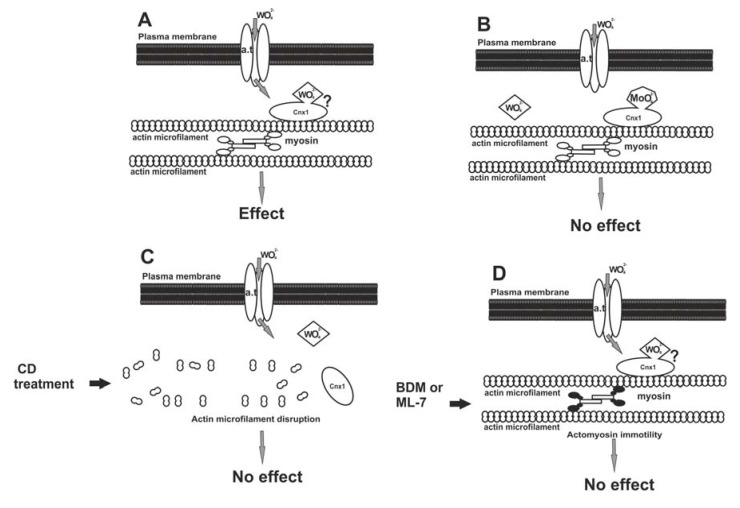
A model for the entry of W in the plant cell, through the pathway of Mo. (**A**) Tungstate anions (WO_4_^2^^−^) pass through the plasma membrane by a transmembrane anion transporter (a.t.) protein. The Cnx1 protein is located close to the plasma membrane due to its binding to cortical actin microfilaments. WO_4_^2^^−^ anions could bind to Cnx1 and be distributed inside the cytoplasm by actomyosin motility. (**B**) Pre-treatment with molybdate results in occupation of the Mo-binding sites of Cnx1, leaving no vacant sites for WO_4_^2^^−^ anions. This prevents the latter from exerting their effect. (**C**) Cnx1 is misplaced due to actin microfilament disruption by cytochalasin D (CD). As a result, WO_4_^2^^−^ anions cannot bind to Cnx1, fail to be distributed inside the cell and do not exert the W effect. (**D**) Inhibition of myosin function by BDM or ML-7 results in arrest of actomyosin-based motility. As a consequence, though bound to Cnx1, WO_4_^2^^−^ anions fail to be distributed. Reprinted from [26], after the model proposed by [93], modified, with kind permission from John Wiley and Sons.

It seems that W may enter the plant cell by exploiting the Mo pathway and binding to the Cnx1 protein at the cell cortex, from where it may be transported in the cytoplasm by actomyosin mobility to exert its deleterious effects (Figure 2A). Should this pathway model be correct, any inhibition of the succession is expected to prevent W to become harmful. Considering this hypothesis, a pharmacological administration involving W, Mo, anti-actin and anti-myosin drugs, singly and in combinations, was staged and the effects were assessed depending on MT morphology [26]. In this research, roots were treated with Na_2_MoO_4_, actin microfilaments were disassembled with cytochalasin D and actomyosin mobility was arrested with BDM (2,3-butanedione monoxide) or ML-7 [1-(5-iodo-naphthalene-1-sulphonyl)-1H-hexahydro-1,4-diazepine] prior to W application. When tissues were pre-treated with Mo, subsequent exposure to W did not affect MTs, apparently due to occupation of all binding sites of Cnx1 molecules by Mo, leaving no vacant sites for W (Figure 2B). When actin microfilaments were experimentally disrupted, the Cnx1 protein was most probably misplaced, not providing the anchor for tungstate anions even though they had entered the cell (Figure 2C). If the actomyosin mobility was inactivated, tungstate anions failed to be distributed intracellularly, though bound to Cnx1, and became ineffective on cortical MTs (Figure 2D). This “protection” of MTs by actin microfilament disruption or actomyosin mobility inactivation further supports the above working hypothesis.

However, in order to “solidify” the above model further experimentation is needed. For example besides microtubules actin microfilaments (the other component of plant cytoskeleton) could be affected by W as well. Indeed, actin microfilaments were almost totally disrupted in W-treated roots of *Pisum sativum*, in parallel with MT disorganization but this effect was correlated with the W-PCD [28], while preliminary experimentation revealed that W treatments induced microfilament bundling [95]. A specific investigation on the effects of W on actin microfilaments is missing and, should that be carried out, it would provide valuable information on the intracellular cascade of events triggered by W toxicity in plant cells.

## 3. Conclusions and Perspectives

As already stated “…it appears that environmental obscurity for W and its compounds has ended and environmental scrutiny has emerged” [1]. In line to this statement, recent advances on the toxic attributes of W in plants revealed a range of adverse effects on morphological, cytological and gene expression levels. W effects on plants could therefore be analyzed under a broadened view that W should not be considered solely a Mo-enzyme inhibitor but a heavy metal having a wide range of effects on plants similarly to other heavy metals [29]. The mechanism by which W is trapped in the roots, the motility of the metal inside the plant body, its cumulative ability and the way it enters in the plant cells are interesting issues to be further examined. Research is also needed in the context of W contamination management and development of phytoremediation technologies. 
